# Genetic Heterogeneity of Self-Reported Ancestry Groups in an Admixed Brazilian Population

**DOI:** 10.2188/jea.JE20100164

**Published:** 2011-07-05

**Authors:** Tulio C Lins, Rodrigo G Vieira, Breno S Abreu, Paulo Gentil, Ricardo Moreno-Lima, Ricardo J Oliveira, Rinaldo W Pereira

**Affiliations:** 1 Programa de Pós-Graduação em Ciências Genômicas e Biotecnologia, Universidade Católica de Brasília, Brasília, DF, Brazil; 2 Programa de Pós-Graduação em Educação Física, Universidade Católica de Brasília, Taguatinga, DF, Brazil

**Keywords:** ethnicity, population structure, ancestry, admixture

## Abstract

**Background:**

Population stratification is the main source of spurious results and poor reproducibility in genetic association findings. Population heterogeneity can be controlled for by grouping individuals in ethnic clusters; however, in admixed populations, there is evidence that such proxies do not provide efficient stratification control. The aim of this study was to evaluate the relation of self-reported with genetic ancestry and the statistical risk of grouping an admixed sample based on self-reported ancestry.

**Methods:**

A questionnaire that included an item on self-reported ancestry was completed by 189 female volunteers from an admixed Brazilian population. Individual genetic ancestry was then determined by genotyping ancestry informative markers.

**Results:**

Self-reported ancestry was classified as white, intermediate, and black. The mean difference among self-reported groups was significant for European and African, but not Amerindian, genetic ancestry. Pairwise fixation index analysis revealed a significant difference among groups. However, the increase in the chance of type 1 error was estimated to be 14%.

**Conclusions:**

Self-reporting of ancestry was not an appropriate methodology to cluster groups in a Brazilian population, due to high variance at the individual level. Ancestry informative markers are more useful for quantitative measurement of biological ancestry.

## INTRODUCTION

The genetic structure of human populations is relevant in epidemiologic studies and can be used as a tool for collecting parental ancestry information in an admixed population. Although the biogeography of some groups is culturally and genetically fixed, other groups have experienced substantial recent admixture with ancestors from widely divergent regions. That is the case in the Brazilian population, which is genetically characterized by differing degrees of admixture of 3 parental populations (European, African, and Native American).^1^^,^^2^

The debate on how genetic studies should be controlled for population stratification has encompassed several methodologies, including self-reported ethnicity and genetic ancestry markers.^3^^–^^8^ Self-reported ancestry has been described as a method that is highly correlated with genetic population structure in well defined, stratified ethnic groups, such as Europeans, Africans, and Asians.^7^^–^^9^ However, in cases of admixed populations, both self-reported ancestry and anthropometric traits used as proxies, such as skin pigmentation, are believed to be unreliable methods of determining ancestry,^3^^–^^5^^,^^8^ which suggests that molecular markers based on genetic clustering should be used to reduce the potential inaccuracies of population stratification.^3^^,^^6^^,^^10^

Many association studies have classified ethnic groups by means of subjective assessment by the interviewer, evaluation of anthropometric traits, genealogical examination, and self-reported ancestry.^11^^–^^15^ However, the recent use of molecular markers to determine genetic ancestry has revealed wide genetic heterogeneity in admixed Brazilians.^5^^,^^16^^–^^22^ One problem in performing association studies of admixed populations that are assessed solely by self-reported ancestry as a proxy of ethnic group is the possibility of spurious association with false-positive or false-negative results.^5^^–^^7^^,^^23^^–^^26^ Thus, the aim of this study was to evaluate the relation of self-reported with genetic ancestry and the statistical risk of grouping an admixed sample by using self-reported ancestry.

## METHODS

### Population sample

Samples were obtained from 189 postmenopausal women (age, 67.77 ± 5.22 years) who had volunteered as part of a healthcare program developed by the Universidade Católica de Brasília, located in the Center-West Region of Brazil (Taguatinga, DF, Brazil). The volunteers answered a lifestyle questionnaire that included a multiple-choice question on self-reported ancestry, based on the method used by the Brazilian Institute of Geography and Statistics (IBGE) national census survey.^27^ All sampled individuals signed an informed consent form, and the research protocol was approved by the University Ethical Committee.

### Assessment of individual genetic ancestry

For assessment of individual genetic ancestry, we selected 13 Ancestry Informative Markers (AIMs) that have differential allele frequencies among European, African, and Amerindian parental populations^28^^–^^30^ (Table 1). The potential informativeness of most of these SNPs was evaluated in a Brazilian population sample,^21^ and a modified method was applied to the use of the current markers. Briefly, genotypic data were obtained by optimized PCR to coamplify DNA fragments in 2 multiplex panels of ancestry informative markers. Later, the PCR-amplified products were purified in an enzymatic treatment with exonuclease I (ExoI) and shrimp alkaline phosphatase (SAP) enzymes to eliminate nonincorporated dNTPs and primers. Finally, the minisequencing reaction was performed using the SNaPshot Multiplex minisequencing kit reaction mix (Applied Biosystems, Foster City, USA), and the products were analyzed on the ABI 3130 XL Genetic Analyzer (Applied Biosystems) in an ABI 3700 POP-6 polymer. Genotypes were called using GeneScan Analysis Software, version 3.7 (Applied Biosystems) and Genotyper version 3.7 (Applied Biosystems). The detailed optimized multiplex single-base extension protocol, with reactant concentration and PCR thermocycling conditions, has been reported elsewhere.^21^^,^^31^^–^^33^

**Table 1.** Allelic frequencies and information on 13 ancestry informative markers for parental populations and studied samples

Locus^ref^	Position	Allele	EUR	AMR	AFR	White	Intermediate	Black
CRH (rs3176921)^30^	8q13.1	G	0.073	0.017	0.682	0.435	0.395	0.767
CYP3A4 (rs2740574)^29^	7q22.1	G	0.958	0.959	0.198	0.485	0.654	0.771
FyNull (rs2814778)^30^	1q23.2	C	0.002	0.000	0.999	0.101	0.237	0.548
LPL (rs285)^30^	8p21.3	G	0.508	0.558	0.029	0.519	0.513	0.226
OCA2 (rs1800404)^30^	15q13.1	G	0.254	0.552	0.885	0.370	0.528	0.717
rs1129038^30^	15q13	C	0.224	0.983	0.995	0.671	0.808	0.871
rs1426654^30^	15q21	C	0.010	0.930	0.970	0.096	0.276	0.758
rs1480642^30^	6q23	C	0.994	0.621	0.106	0.788	0.679	0.435
AT3 (rs3138521)^28^	1q25	Insertion	0.282	0.061	0.858	0.354	0.304	0.597
rs736556^30^	7p15	C	0.244	0.018	0.939	0.242	0.313	0.519
rs3768641^30^	2p13	G	0.923	1.000	0.010	0.715	0.725	0.577
rs1871534^30^	5p15.2	G	0.019	0.000	0.960	0.029	0.049	0.250
rs4766807^30^	12q24.2	A	0.622	0.948	0.030	0.559	0.542	0.429

European (EUR), African (AFR) and Native American (AMR).

### Statistical analysis

Allelic frequencies were obtained by direct counting, along with pairwise population Fixation index (F_ST_) analysis, which was performed using GenAlex software.^34^ The F_ST_ measures population differentiation based on the heterozygosity of genetic polymorphism data is calculated using the formula, F_ST_ = (H_T_ − H_S_) × H_T_^−1^, where H_T_ is the expected heterozygosity in the total population and H_S_ is the observed heterozygosity in a subpopulation.^35^ The fixation index can range from 0.0 (no differentiation) to 1.0 (complete differentiation) and theoretically varies from little (0.0 to 0.05) to moderate (0.05 to 0.15), great (0.15 to 0.25), or very great genetic differentiation (>0.25).

Estimation of individual genomic ancestry was performed using an algorithm based on maximum likelihood estimation (MLE). Briefly, the log likelihood function is maximized for the admixture parameter of up to 3 parental populations using a priori known allele frequencies and estimates the individual ancestry probability from a predetermined number of analyzed genotypes of an admixed individual. The MLE approach was implemented in the software program IAE3CI; the detailed statistics have been described elsewhere.^36^^,^^37^

Basic descriptive statistics and 1-way analysis of variance (ANOVA) with the post-hoc Games-Howell test to adjust for unequal variances were used to determine the relation between genetic ancestry distribution and self-reported ancestry groups. A *P* value of 0.05 or lower was considered statistically significant. Statistical analysis was performed using SPSS software version 13 (SPSS Inc., Chicago, IL, USA).

## RESULTS

A total of 192 participants completed the study, but only 3 were of self-reported Amerindian ancestry. Due to the lack of statistical power, these 3 women were not considered in the analysis, and 189 participants remained for study, as previously described. The questionnaire responses indicated that the sampled population had similar prevalences (41.8%) for 2 groups, white and intermediate (ie, any sort of admixture). Blacks represented 16.4% of the sample. No individual reported Asian descent in the present research.

Allelic frequency for the 13 genotyped SNPs is described in Table 1, along with the frequencies of the parental populations. Using Wright’s scale of genetic differentiation, F_ST_ analysis revealed little difference between the white and intermediate groups (F_ST_ = 0.022), a moderate difference between the intermediate and black groups (F_ST_ = 0.138), and great differentiation between the black and white groups (F_ST_ = 0.225); all *P*-values were significant.

The genetic ancestry of each self-reported ancestry category and the total sample was estimated (Table 2). The range of individual ancestry for the 3 parental genomes within each self-reported ancestry category is depicted in a box plot (Figure). The 3 self-reported categories had overlapping ranges for each parental ancestry. For example, with regard to European ancestry, there were individuals in all 3 self-reported categories within the range of 0.41 to 0.78 for the ancestry proportion. For African ancestry, this overlap ranged from 0.19 to 0.48, and for Amerindian ancestry the range was from 0 to 0.42 (Figure). For instance, an individual in the black group had 78% European ancestry and 22% African ancestry (sample 181; Figure).

**Figure.**
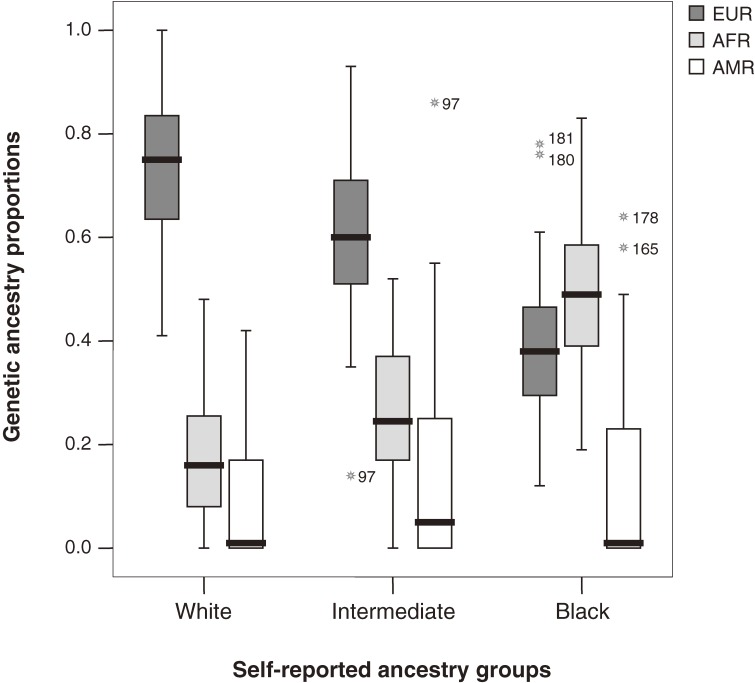
Boxplot of genetic ancestry estimates of European (EUR), African (AFR), and Amerindian (AMR) proportions among the 3 self-reported groups. *outliers.

**Table 2.** Genetic ancestry estimates among self-reported ancestry groups, by skin color

Self-reported ancestry	European		African		Amerindian	
		
	AV ± SD	VAR	AV ± SD	VAR	AV ± SD	VAR
White (*n* = 79)	0.738 ± 0.135	0.018	0.172 ± 0.134	0.018	0.090 ± 0.120	0.014
Intermediate (*n* = 79)	0.615 ± 0.140	0.020	0.256 ± 0.142	0.020	0.129 ± 0.168	0.029
Black (*n* = 31)	0.387 ± 0.164	0.027	0.472 ± 0.158	0.025	0.141 ± 0.189	0.035
Total (*n* = 189)	0.629 ± 0.187	0.035	0.254 ± 0.174	0.031	0.117 ± 0.155	0.024

Average values (AV), standard deviations (SD), and variance (VAR).

One-way ANOVA for comparison of means in conjunction with the Games-Howell post-hoc test revealed significant mean differences among self-reported groups for European and African ancestries, but not for Amerindian ancestry (Table 3). Although significant, the confidence interval showed that, for European and African ancestries, the average range of the boundary limits was 0.14, which indicates that the probability of spurious findings (type 1 error) arising simply by chance was 14% higher; the usual result is 5% (*P* = 0.05).

**Table 3.** Comparisons of genetic ancestry among groups, by self-reported skin color. The mean difference was considered significant at *P* ≤ 0.05

Dependent variable	(i) Group	(j) Group	Mean difference (i − j)	Standard error	*P*-value	95% Confidence interval	

						Lower bound	Upper bound
EUR	White	Intermediate	0.122	0.022	<0.001	0.070	0.174
		Black	0.351	0.033	<0.001	0.271	0.432
	Intermediate	White	−0.122	0.022	<0.001	−0.174	−0.070
		Black	0.229	0.034	<0.001	0.148	0.310
	Black	White	−0.351	0.033	<0.001	−0.432	−0.271
		Intermediate	−0.229	0.034	<0.001	−0.310	−0.148

AFR	White	Intermediate	−0.078	0.022	0.001	−0.130	−0.026
		Black	−0.300	0.032	<0.001	−0.377	−0.222
	Intermediate	White	0.078	0.022	0.001	0.026	0.130
		Black	−0.221	0.033	<0.001	−0.300	−0.143
	Black	White	0.300	0.032	<0.001	0.222	0.377
		Intermediate	0.221	0.033	<0.001	0.143	0.300

AMR	White	Intermediate	−0.041	0.023	0.187	−0.097	0.014
		Black	−0.051	0.037	0.349	−0.141	0.038
	Intermediate	White	0.041	0.023	0.187	−0.014	0.097
		Black	−0.010	0.039	0.964	−0.105	0.084
	Black	White	0.051	0.037	0.349	−0.038	0.141
		Intermediate	0.010	0.039	0.964	−0.084	0.105

European (EUR), African (AFR), and Native American (AMR) proportions.

## DISCUSSION

The Federal District is a modern urban center and the capitol of Brazil. It has a population of migrants from several regions of Brazil. The 2007 National Household Sample Survey reported a distribution of self-reported ancestry in the Federal District that was very similar to that of the present study, differing only in the prevalence of blacks.^27^ The inferred ancestry estimated here is comparable to those of other published studies of the Center-West Brazilian population,^17^^,^^21^ with only slight differences in European and African ancestry proportions, which were probably due to sampling issues.

In this study, the statistical power of the 13 AIM panel might have been insufficient to accurately assess ancestry in an admixed population.^38^ However, when the population ancestry estimates, standard deviations, and variances of the present study were compared with those of a different sample of Center-West Brazil that was assessed using a set of 28 ancestry markers,^21^ the values did not statistically differ between samples (*P* = 0.49), especially with regard to individuals of Amerindian ancestry (0.118 ± 0.149, variance 0.022, in the earlier sample).^21^

Allelic frequency and F-statistic estimates significantly differed among groups. It is noteworthy that the differences in allelic frequencies between the corresponding ancestry-related populations (ie, European versus white Brazilians and African versus black Brazilians) were remarkably divergent. This was the case for CYP3A4 in EUR-white (δ = 0.473) and for rs1871534 in AFR-black (δ = 0.710), which highlights the admixture among these groups. The proportions of genetic ancestries in the intermediate group were similar to those of the total sample and differed only in variance. Therefore, the range amplitude and variance of ancestry at an individual level were too large for self-reported ancestry to be considered a suitable proxy for homogenic clustering, although the differences in their means were statistically significant.

In addition, we observed an overlap in the range of genetic ancestry values among groups, which suggests that individuals with the same proportions of admixture could include themselves in any ethnic category. The confidence interval revealed that the risk of this occurring simply by chance was 14%, considering the European and African ancestry estimates. It is worth mentioning that the group self-reported as black had a proportion of non-African ancestry exceeding 53%. In previous studies of the Brazilian population, African ancestry did not exceed non-African ancestry, but a large proportion was observed in a sample of the rural community of the Southeastern Brazilian state of Minas Gerais (48% non-African ancestry)^5^ and in an urban population sample from Rio de Janeiro (49% non-African ancestry).^39^ The intermediate group described here had estimates closer to those of the white group, as was the case for the urban population of Rio de Janeiro.^39^ Alternatively, in a study of a sample from a rural community,^5^ the intermediate group was closer to blacks, revealing an important issue, namely, that groups with equal self-reported proportions can have different genetic ancestry profiles, especially if the samples are from communities with different levels of urbanization. Those features were also demonstrated in other Brazilian population samples that assessed ancestry by using maternal (mtDNA) and paternal (Y-chromosome) molecular markers.^20^^,^^22^^,^^40^

A related study of Puerto Ricans who self-classified ancestry/color groups found statistically significant differences in genetic ancestry among 3 groups (blanco, trigueño, and negro).^41^ The results of that study can be compared with the categories in the present study. An overlapping range in ancestry estimates was also reported, in which the distribution of African ancestry overlapped across 12% for all 3 color categories (range of ancestry estimates: 0.27–0.35). In the present sample, this range was considerably higher (0.19–0.48), which encompassed 48% of the sample. For European ancestry, the overlap accounted for 63% of the sample in a range between 0.41–0.78, while for Amerindian ancestry, 95% of individuals were in the overlapping range (0.0–0.42) for all 3 categories.

The reliability of self-classification can be poor, even among a proband and siblings,^4^ in which the family history would be assumed to be more reliable. In the same way, interviewers might misclassify an individual for whom they do not know the ancestral family history. Indeed, different interviewers have classified the same individual into different groups.^23^^,^^42^ These examples illustrate how self-identified ethnicity might not be sufficiently accurate for use in biomedical research, as it is primarily a sociocultural construct.^42^ Considerable variation exists because ethnicity is essentially constructed under social circumstances that consider many cultural traditions.^1^^,^^42^^,^^43^ In admixed populations, individuals might feel that they belong to a certain ethnic group for cultural reasons or beliefs; however, their genealogy might consist of an unknown admixture.^1^^,^^2^ Although our sample comprised only women, we did not evaluate such effects on self-declared ancestry, as it may have more sociological than biological meaning.^42^^,^^43^ From a sociological and anthropological point, a person’s biological ancestry might have no relationship to their self-identification with a cultural group, but such ancestry might be of great importance in clinical research.

In conclusion, determination of ethnicity based on self-reported ancestry is vulnerable to misclassification and should be avoided in scientific research. Therefore, the concept of ethnic group and self-declared ancestry are not synonymous in biomedical research and must be replaced with scientific measurements that have biological meaning, such as individual ancestry estimated by DNA markers.^5^^,^^24^^,^^44^ Several strategies can be effective in controlling heterogeneity equivalence. For example, individual ancestry estimates can be used to match admixed case-control groups.^45^ They can also be used in cross-sectional studies as covariates to adjust for a population stratification effect.^24^^,^^31^^,^^33^ The use of ancestry informative markers to estimate individual ancestry is an effective and reliable solution to correct the effects of heterogeneity.
